# Assessment of heated tobacco products in Cairo and Giza at points of sale: Availability, advertisement, and promotion

**DOI:** 10.18332/tpc/200819

**Published:** 2025-02-26

**Authors:** Amira Haridy, Heba AlSawahli, Eman H. Elsebaie, Fatimah El Awa, Nibras ELhag Arabi, Abla Elalfy, Randa Abou El Naga

**Affiliations:** 1 Egyptian Members’ Association, Royal College of Pediatrics and Child Health, Egypt; 2 World Health Organization Egypt Country Office, Cairo, Egypt; 3 Faculty of Medicine, Cairo University, Giza, Egypt; 4 Armed Forces College of Medicine, Cairo, Egypt; 5 World Health Organization Regional Office for the Eastern Mediterranean, Cairo, Egypt; 6 Ministry of Health and Population, Cairo, Egypt

**Keywords:** tobacco advertisement, HTPs, point of sale, tobacco promotion, sale to minors

## Abstract

**INTRODUCTION:**

Heated tobacco products (HTPs) are readily available at diverse points of sale (POS) in Egypt. This study aims to assess these advertisements and promotions to provide evidence for policymakers on the need for tobacco control law amendments and enforcement in Egypt.

**METHODS:**

A cross-sectional descriptive study was conducted in Cairo and Giza governorates in 2022 through a convenience sample to collect data from 150 POS. The study's data collection tools assessed the availability, display, advertisement, and promotion of HTP at each site.

**RESULTS:**

Price promotions were available at 18% of the visited sites, ranging between bundles and promo code discounts; 75% of the points of sale had some type of advertisement, either inside (67.3%) or outside (36%), stating that HTP are less harmful than traditional cigarettes because they do not burn. HTP display was commonly around the cashier area (87.3%), followed by candy and gum (80.7%) or soda, ice cream, or coffee machines (66%).

**CONCLUSIONS:**

The reported advertisement and promotion of HTP at POS and their sale to minors violate the National Tobacco Control Law 52/1981. These violations risk the health of the youth. We call on policymakers to explicitly ban all sorts of advertisement and promotion of tobacco products at POS, and enforce the ban of sale to minors (under 18 years).

## INTRODUCTION

Tobacco control in Egypt is a multilayered challenge. Contrary to the global trend, tobacco use is increasing in Egypt^1^, especially with the emergence of heated tobacco products (HTPs). The prevalence of tobacco use, according to the 2017 STEPS survey, was 22.7%, with a higher percentage among males (43.4%) than females (0.5%)^2^; 18.1% of boys and 8.2% of girls (aged 13–15 years) used tobacco^3^. There are uncertainties about the data reliability about tobacco use in women because of the cultural stigma of smoking among women in Egypt^4^. Studies from neighboring Arab countries showed that during household surveys, women tend not to disclose their smoking habits in the presence of male family members, as it is perceived as a shameful behavior among them^5,6^. More males (62.9%) are expected to use tobacco in 2025^7^.

Tobacco is a major risk factor for non-communicable diseases (NCDs), including cardiovascular diseases, chronic respiratory diseases, and cancers that cause 86% of deaths in Egypt^8^. Tobacco-related illnesses kill 95000 Egyptians every year, mainly before the age of 70 years^9^. Since these illnesses require long-term, expensive medical treatment, concerned citizens spent around EGP 4.5 billion (about US$ 245 million) in out-of-pocket healthcare expenditures. Tobacco costs the country EGP 89.8 billion in 2017, amounting to 2.1% of the country’s GDP in the same year.

Egypt ratified the WHO Framework Convention on Tobacco Control in 2005 and has tobacco control laws and policies that comply with the MPOWER policies^10^. However, the financial and human resources for enforcement are scarce, besides the low political commitment and tobacco industry interference^11^. Law 52/1981 prohibits all forms of advertisement and promotion of all tobacco products. The penalty for noncompliance could be a year in jail or paying at least 1000 EGP and not more than 5000 EGP. The law also bans tobacco sales to people aged <18 years through its amendments by Law 85/2002, with a fine between 100 EGP and 1000 EGP on vendors who do not comply. The industry hindered an inclusive ban on promoting tobacco a few years ago^12^; and works on the ground through price discounts and promotional allowances to make tobacco products easier to notice and buy at the POS^13^.

Evidence shows that the placement and presentation of tobacco products to customers could significantly make them decide to buy cigarettes even if they did not intend to buy them initially^14,15^. Moreover, it makes quitting more difficult as it stimulates cravings. Notably, tobacco products influence young people and females the most^16,17^. The challenge in Egypt is augmented by the relatively lax legal environment and not applying control measures and industry tactics to attract the young population and females.

Heated tobacco products (HTPs) came to Egypt in 2018, and Ministerial Decree 79/2021 required the application of a health warning for their packages^18^. The tobacco industry markets HTPs, claiming that they are a safer option than traditional cigarettes and often use health professionals to make them acceptable. The industry refers to HTPs as ‘smoke-free’ products to confuse officials and the public to escape tobacco control laws^19^. This study aims to investigate and document the use of POS to promote HTPs in violation of national laws in Egypt.

## METHODS

### Design, setting, sample, and tools

A cross-sectional descriptive study was conducted in Cairo, the capital city, and urban areas of the Giza governorates of Egypt, which are closely located and highly connected through roads and metro lines. Cairo neighborhoods were classified according to administrative classification into north, south, east, and west. Urban areas of Giza were classified into old neighborhoods that are closely connected to Cairo and new ones, which are mainly Sheikh Zayed and ‘6th of October’ areas. The sample selection was based on the multistage proportionate stratified cluster random technique. The strata were Cairo and urban areas of Giza Governorates (primary sampling units), and the sample was distributed proportionally among them. Then, each governorate was stratified into districts (secondary sampling units). Sub-districts formed the tertiary sampling units. The fourth-level sampling units were the streets (data collectors had Google Maps for each subdistrict). POS were considered the end sampling units. At each level of the sampling methodology, simple or systematic random sampling techniques were used for randomization and representation of the selected sample. For districts and subdistricts, cluster sampling was performed using the rule of thumb in proportion to the total number of clusters. As a complete listing of POS in the survey area was not available, systematic random sampling was used in the selection (every other POS in every other street). A total of 150 POS in 17 neighborhoods were observed.

The following formula was used to calculate the sample size of this cross-sectional survey:

n = [DEFF×N×p(1-p)]/ [(d^2^/Z^2^_1-α/2_×(N-1)+p*1-p)]

Assuming a 95% confidence level, 5% level of significance, an estimated similar proportion of 50%, and design effect of 2, the minimum required sample size for this study was 135 places. We added 10% (n=14) to accommodate for possible missing data, so finally 150 places were included.

The data collection tool of the study was an observation list on POS, which was adapted from the TAPS compliance assessment guidance of the Johns Hopkins Bloomberg School of Public Health, the Campaign for Tobacco-Free Kids, and the International Union against Tuberculosis and Lung Disease^20^.

In January 2022, the study coordinator created a checklist to be accessed online by the data collectors and trained them on all the study details and how to enter the data on their phones. Variables were the address and type of the selected POS, availability of HTP devices and cigarettes, presence of HTP price promotions or bundles, presence of HTP advertisements, whether inside or outside the POS, and display of the HTP at the POS and observing the sale to minors.

At the end of the training, an inter-observational assessment was performed to verify the obtained data. Retailers were not asked to give formal consent to avoid tempting them to give an ideal answer.

### Data collection process

Over three weeks between February and March 2022, a team of six data collectors visited 150 POS ranging between kiosks, smoking shops, malls, and grocery stores in the selected neighborhoods (Supplementary file). For each POS, the assigned data collector conducted the observation, and they spent around 20–30 minutes at the POS observing. The field researchers directly entered the collected data into Google Forms at the POS. By the end of each week, the study coordinator did a random check of 10% of the records to confirm data completeness.

### Statistical analysis

Univariate analysis was done using the Statistical Package for Social Science Software (SPSS, version 22.0, IBM) for quantitative variables, to summarize the data.

## RESULTS

### Distribution of observed POS

The total number of visited and assessed POS was 150, distributed over 17 neighborhoods in Cairo and urban areas of Giza. The distribution and type of POS are given in Tables 1 and 2.

**Table 1:** Distribution of the visited points of sale in Cairo and urban Giza (N=150)

*Governorate*	*n*	*%*
Cairo (East)	39	26.0
Cairo (West)	9	6.0
Cairo (South)	28	18.7
Cairo (North)	36	24.0
Giza (old neighborhoods)	18	12.0
Giza (new neighborhoods)	20	13.3

**Table 2:** Type of visited point of sale (N=150)

*Type of point of sale*	*n*	*%*
Shop/booth inside a shopping mall	9	6
Street kiosk beside a gathering spot (shopping malls, clubs, coffee shops)	97	64.4
Convenience store inside gas station	24	16
Separate store with a franchise brand	6	4
Other (convenience stores, supermarkets, etc.)	14	9.3

### Heated Tobacco Products (HTPs) availability

Among the visited shops, IQOS was present at 74% of them. GLO was present at 37% of POS. IQOS devices were available at 16% of POS, while GLO devices were available at only 9% of them, as shown in Table 3.

**Table 3:** Availability of HTPs and their types in the visited POS (N=150)

*Availability*	*IQOS device*		*IQOS sticks*		*GLO device*		*GLO sticks*	
	*n*	*%*	*n*	*%*	*n*	*%*	*n*	*%*
Yes	24	16	111	74	14	9.3	55	36.7
No	126	84	39	26	136	90.7	95	63.3

### HTP advertisement

At 75% of visited POS, there was at least one type of advertisement for HTPs. In 67% of POS, there were branded advertisements inside the shops, and in 36%, there were advertisements outside (Table 4). Advertisements were in the form of posters, signs, and hangers. Some POS had their shop sign showing the brand name of tobacco products. Two malls had branded booths dedicated to HTPs inside them. Data collectors noted that ads stated that HTP are less harmful than traditional cigarettes because they do not burn. As part of the advertisement process, clerks were available to demonstrate to the customers how to use the devices and explain their ‘potential health benefits’. Other advantages reported included the improvement of the sense of smell and taste as a major ‘benefit’ opposite to traditional cigarettes.

**Table 4:** Rate of HTP advertisement and its types (N=150)

*HTP advertisement*	*n*	*%*	*Type of advertisement*	*n*	*%*
Total POS with HTP advertisement	113	75.3	Branded advertisement outside the POS	54	36
			Branded advertisement inside the POS	101	67.3
			Branded booth inside a shopping mall	2	1.3
Total POS without HTP advertisement	37	24.7			

### HTP display and placement at visited POS

The study found that retailers displayed HTP primarily around the cashier area (87.3%), followed by beside candy and gum (80.7%), or soda, ice cream, or coffee machines (66%). About 24.7% of POS placed them beside children’s toys.

### HTP price promotions

Price promotions for HTPs were observed at 18% of the visited POS (Table 5). The most common promotion strategy was offering free packets of sticks upon device purchase (77.8%). For instance, IQOS’s ‘discovery bundle’ offered three packets of sticks upon buying one device. Another strategy was direct marketing by current users to other potential users from their circle. Using a specific code on the current user’s device, they invite new users to receive a reward of 300 EGP discount (about US$15 at the time of the study) which was about 20% of the price at the time of the study. The same reward was given to the current user as a discount voucher.

**Table 5:** HTP price promotion and its types (N=150)

*Promotion*	*n*	*%*	*Types of promotion*	*n*	*%*
Total POS with HTP promotion	27	18	Bundles: buy the device and get free packets of sticks (different bundles based on device price)	2	77.8
			Promo code discount	4	14.8
			Bundle and promo code discount	2	7.4
Total POS with No HTP promotion	123	82			

### HTP sale to minors

None of the vendors at the observed POS asked for an ID before the sale of HTPs, as reported by the data collectors who stayed for 20–30 minutes at each POS. Young people were allowed to buy HTPs from 100% of the visited POS.

## DISCUSSION

The study found that three-quarters of visited points of sale had some sort of advertisement for heated tobacco products. Price promotions were also present in almost 20% of the studied locations. These practices violate the Egyptian Tobacco Control Law number 52/1981 whose Articles 5 and 6 prohibit all types of advertisement and promotion of tobacco products. Although Law 52/1981 and its amendments by Law 85/2002 ban the direct advertising of tobacco, it does not explicitly ban display, indirect advertising, promotion, and sponsorship activities. The law does not mention the display of tobacco products at the POS specifically, thus the tobacco industry uses this legal loophole to promote its products. In the United States in 2021, the tobacco industry spent nearly 80% of its marketing budget in the retail environment^14^. This reflects the high importance of the end sites of the supply chain at the retailers and POS.

The reported false information in almost all ads about the relative less damage by HTP than traditional cigarettes is a further alarming finding that should be considered in the tobacco control law. The tobacco industry has been relentlessly using the tactic of ‘harm reduction’ to attract more customers and retain customers through their addictive products^21^. It funds research studies to bring scientific evidence to greenwash their motives^22^. Accordingly, such claims should be included in the category of false and deceiving words like ‘light’ or ‘less tar’ and the same penalties should be applied in Egyptian law.

The display of tobacco products is in itself an advertisement and promotion tool^23^. There is plenty of evidence about the positive association between exposure to the promotion of tobacco at POS and smoking. Impulse purchases and normalization of tobacco use are found to decrease in countries where tobacco display is banned^24,25^. Moreover, tobacco companies use the retail environment to attract new customers and maintain current users through product placement^26,27^. Power walls and standalone displays are usually behind the cashier counter or beside everyday items and children’s products such as batteries, magazines, soda, and candy products. Such placement normalizes the presence of tobacco products and their use, increases the duration of exposure to the advertisement, and makes these products socially acceptable and accessible. This tactic desensitizes the public, especially youth, about the deleterious health consequences of tobacco use^28,29^. An experimental study among adolescents in the USA showed that hiding the tobacco power wall significantly reduced the odds of participants’ susceptibility to smoking^30^. A repeat cross-sectional study among never-smoking adolescents in the UK showed a significant reduction in smoking susceptibility following complete and partial bans on display^31^.

HTPs come under the same regulation of tobacco products as per the Ministerial Decree 79/2021.

Despite being prohibited by Egyptian law, HTP promotion was observed in 18% of the studied POS. This is a common global challenge where legislation does not comprehensively address this relatively new product. Tobacco companies tend to promote the heating device to bypass the law^32^. In this study, data collectors documented that at particular POS, clerks were available to present the product. PMI used the same approach in South Korea and Canada where professional presenters at IQOS stores provided information about the device and invited the customers to register at websites to get discounts and extended warranty^33^.

Egyptian tobacco control law does not ban the display and visibility of tobacco products at POS. Therefore, it is common to find storefronts freely displaying a variety of tobacco products. In this study, HTPs were found to be placed in accessible observed areas at the POS, mainly around the cashier and beside the candy area in more than 80% of the visited spots. This is quite common in the Eastern Mediterranean Region, where only six countries ban the display of tobacco products at POS^34^.

It is worth mentioning that the visited points of sale were located within densely populated Cairo and Giza governorates which are residences for more than 20 million people. Taking into consideration the demographic characteristics in Egypt, with 43.7% of the population aged ≤19 years^35^. Ban of sale to those under aged (<18 years) was not observed by any of the visited POS, which constitutes a serious threat to health, especially to children and youth.

### Limitations

The study has some limitations. First, we only covered a limited area, which does not allow us to extrapolate our findings to the whole country. Second, we could not survey young people about their knowledge and motives for buying HTP, and how advertisement and promotion influence their decisions.

## CONCLUSIONS

The study provides evidence for the high need to revisit the existing tobacco control legislation, to be reviewed and amended regarding the missed and ambiguous statements concerning the ban on tobacco product display and indirect advertisement. It should comprehensively ban all forms of display, advertisement, and promotion following Article 13 of the FCTC. Furthermore, the government of Egypt should allocate more financial and human resources to enforce the tobacco control law including the bans on tobacco advertisement, promotion, and sponsorship (TAPS).

## Supplementary Material



## Data Availability

Data are available from the authors on reasonable request. The data set of the visited points of sale can be accessed by writing to alsawahlih@who.int.
