# Supplementary material for: Assessment of heated tobacco products in Cairo and Giza at points of sale: Availability, advertisement, and promotion
Source: Tob Prev Cessat. 2025 Feb 26;11:10.18332/tpc/200819. doi: 10.18332/tpc/200819 (PMC11863967; doi:10.18332/tpc/200819)
Supplement: Supplementary file 1 [file TPC-11-15-s1.pdf]

Supplementary File

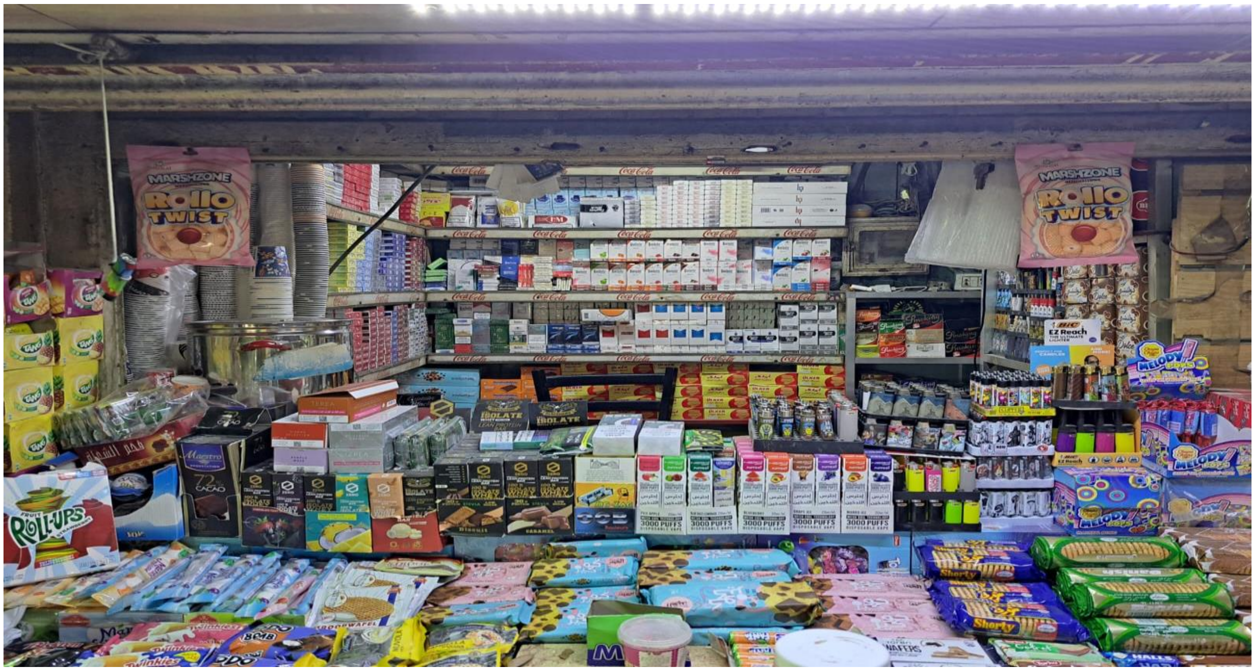

Figure 1Tobacco products placed beside candy and gum at a street kiosk in Cairo

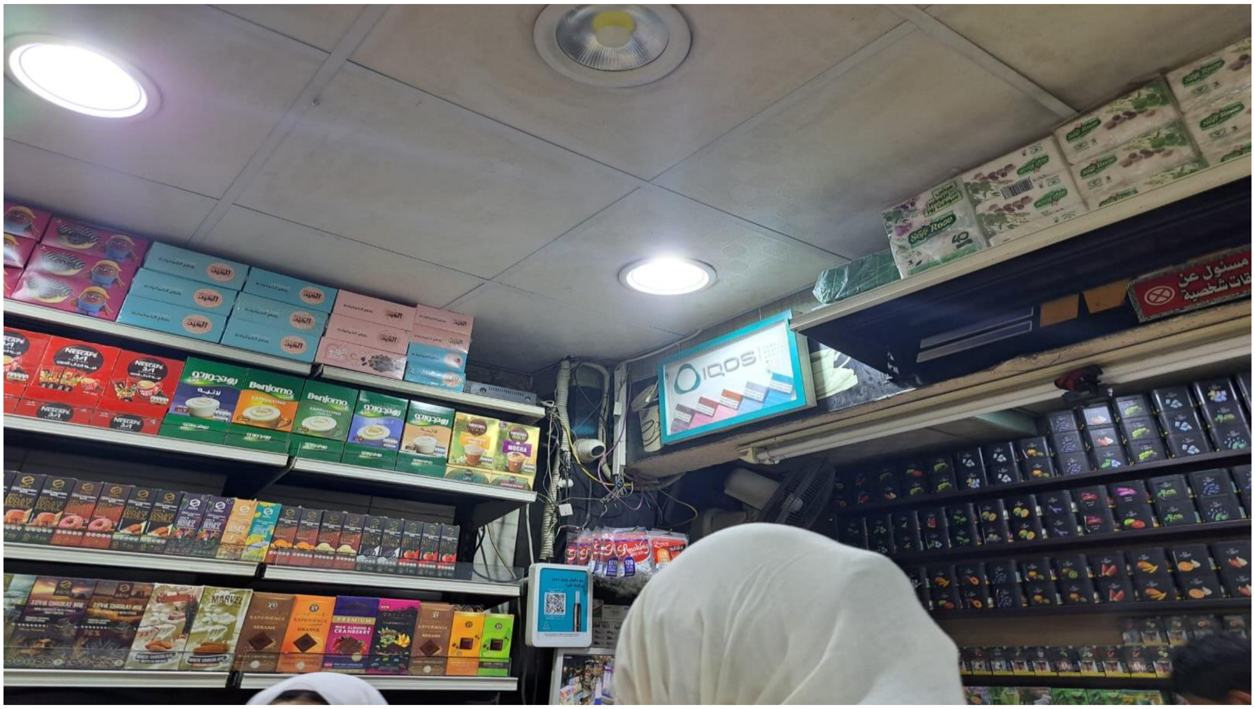

Figure 2 Branded advertisement and display of HTP at a street kiosk in Cairo

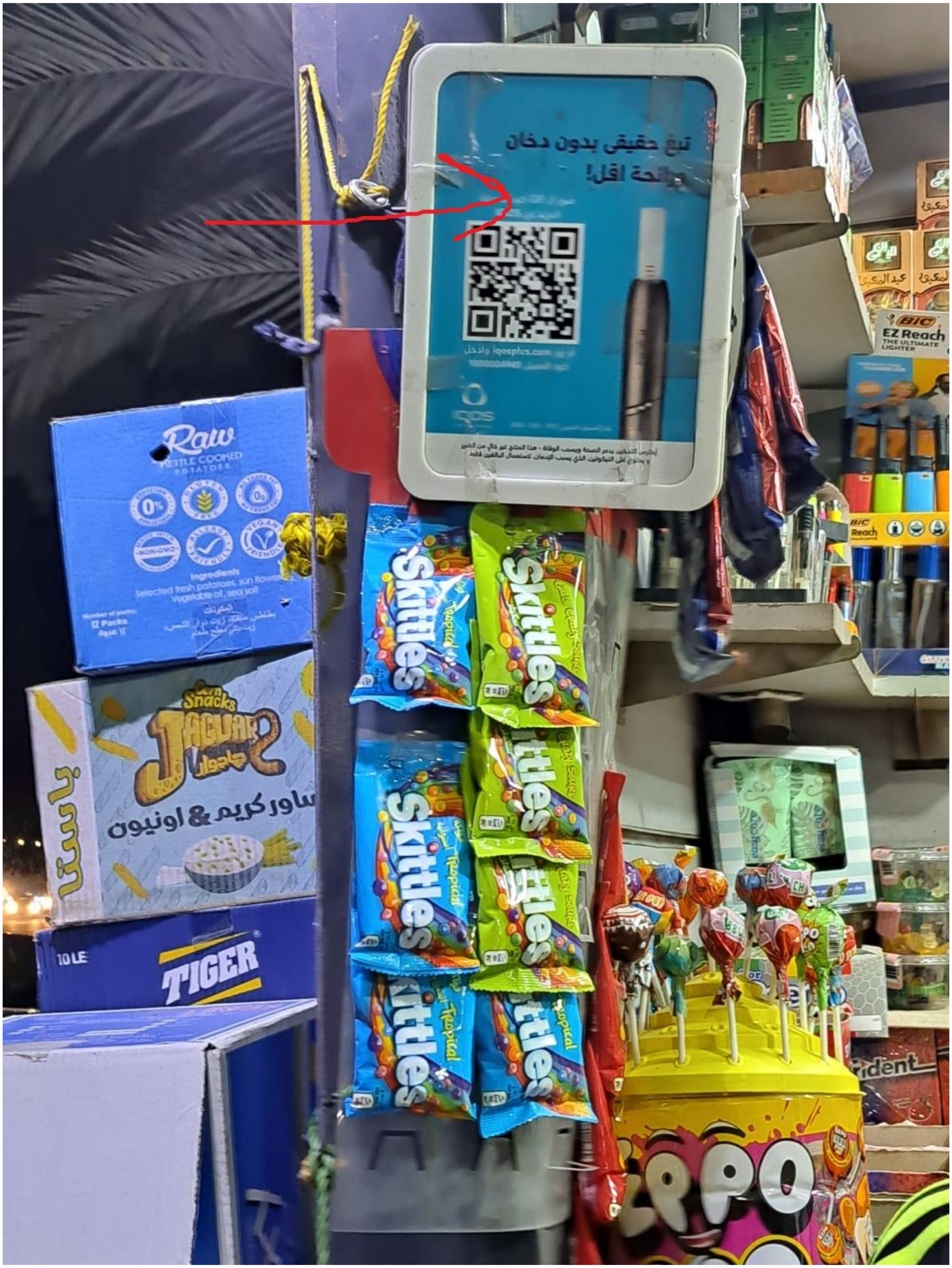

Figure 3 Branded advertisement of IQOS: “True tobacco, smokeless, with less smell!”- MARKED

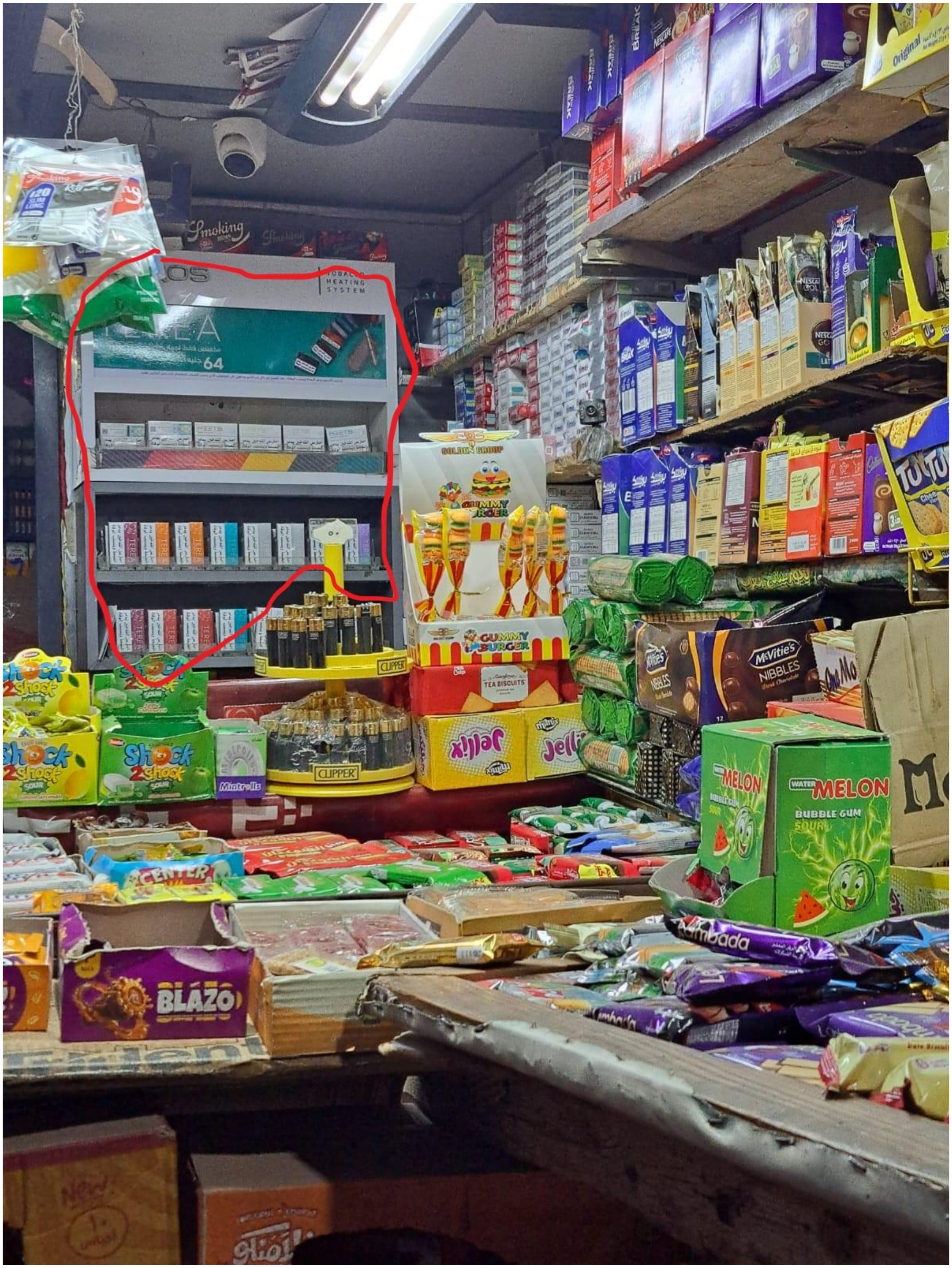

Figure 4 Display of HTP and placement near candy at a street kiosk in Giza – MARKED

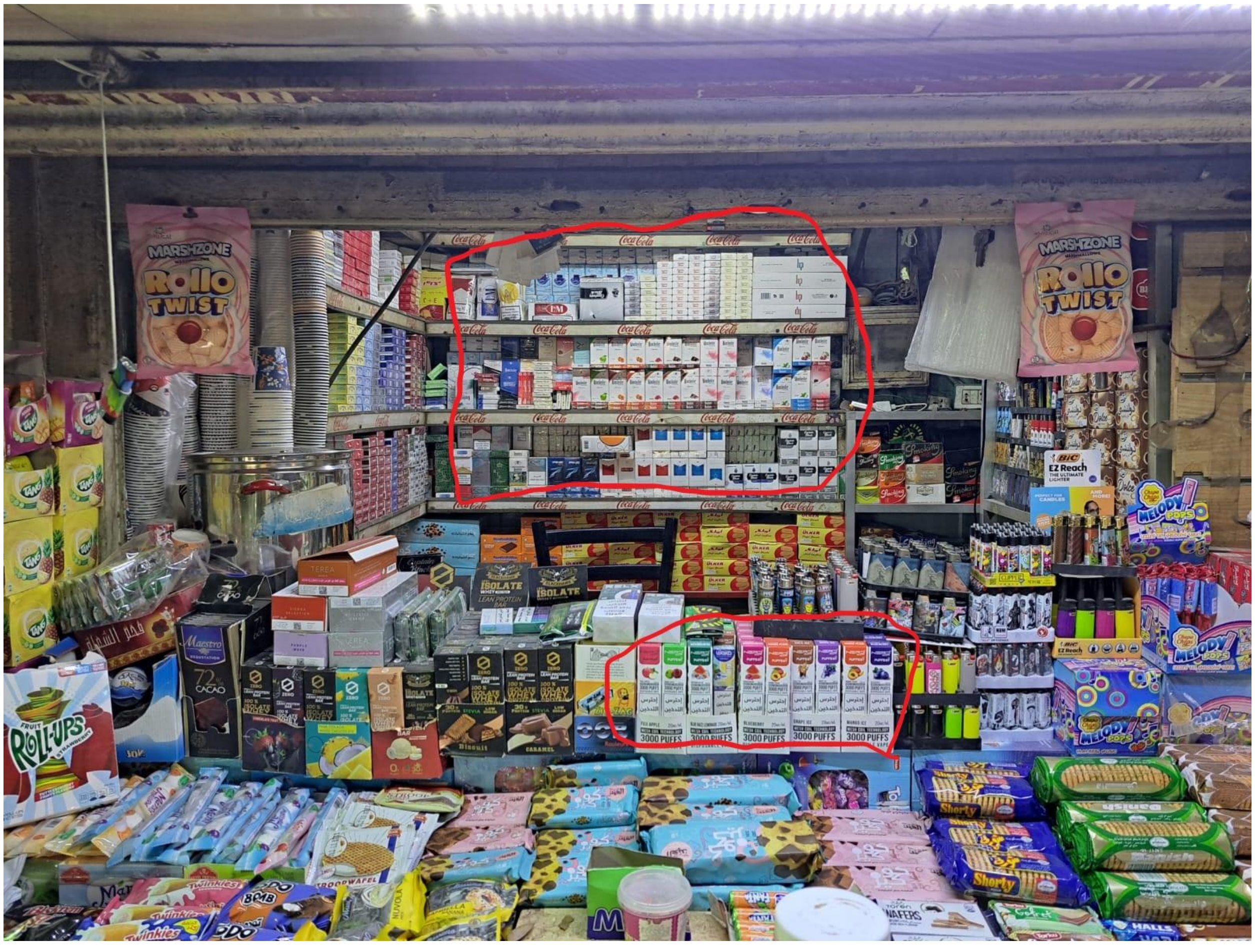

Figure 5 Tobacco products placed beside candy and gum at a street kiosk in Cairo- MARKED

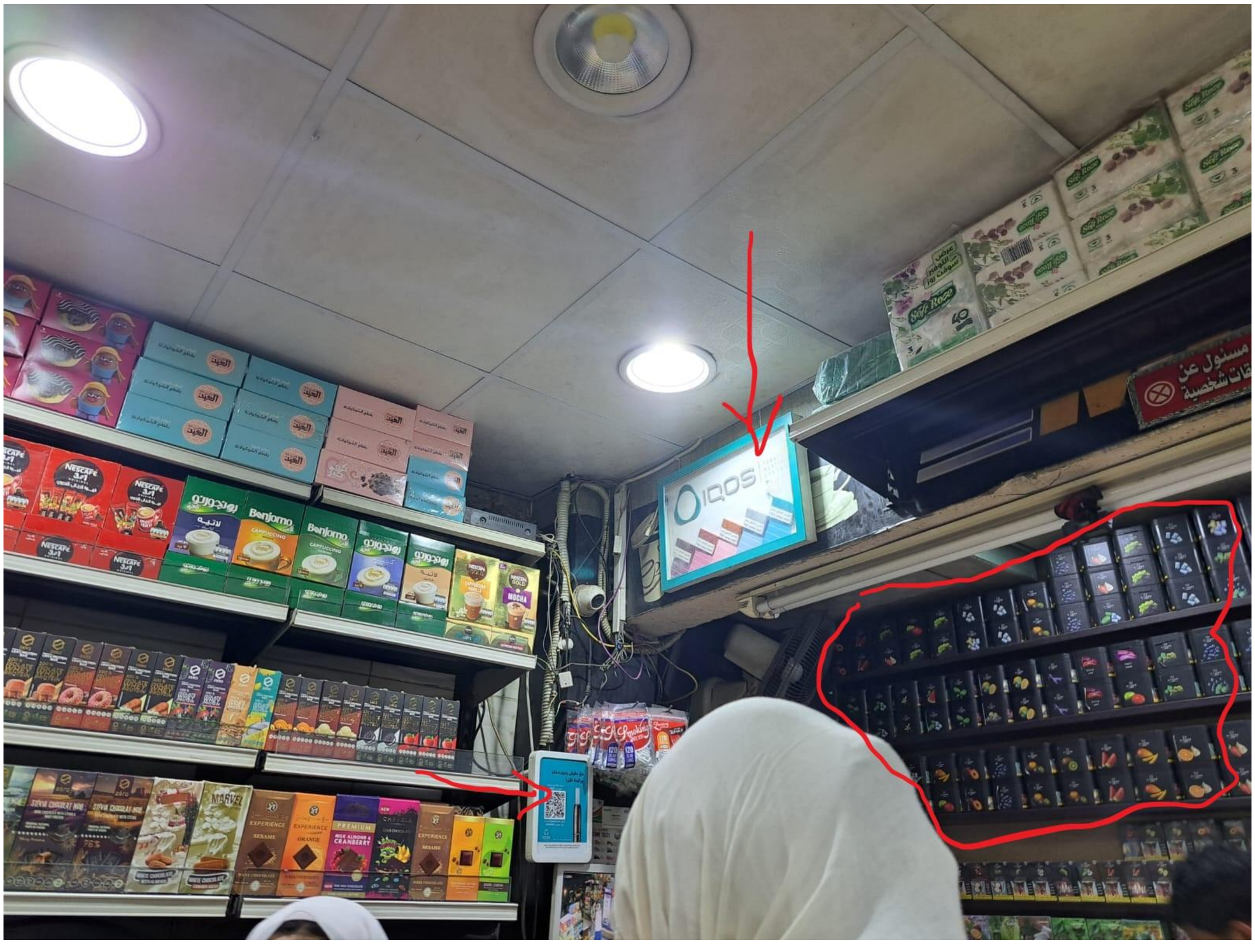

Figure 6 Branded advertisement and display of HTP at a street kiosk in Cairo- MARKED

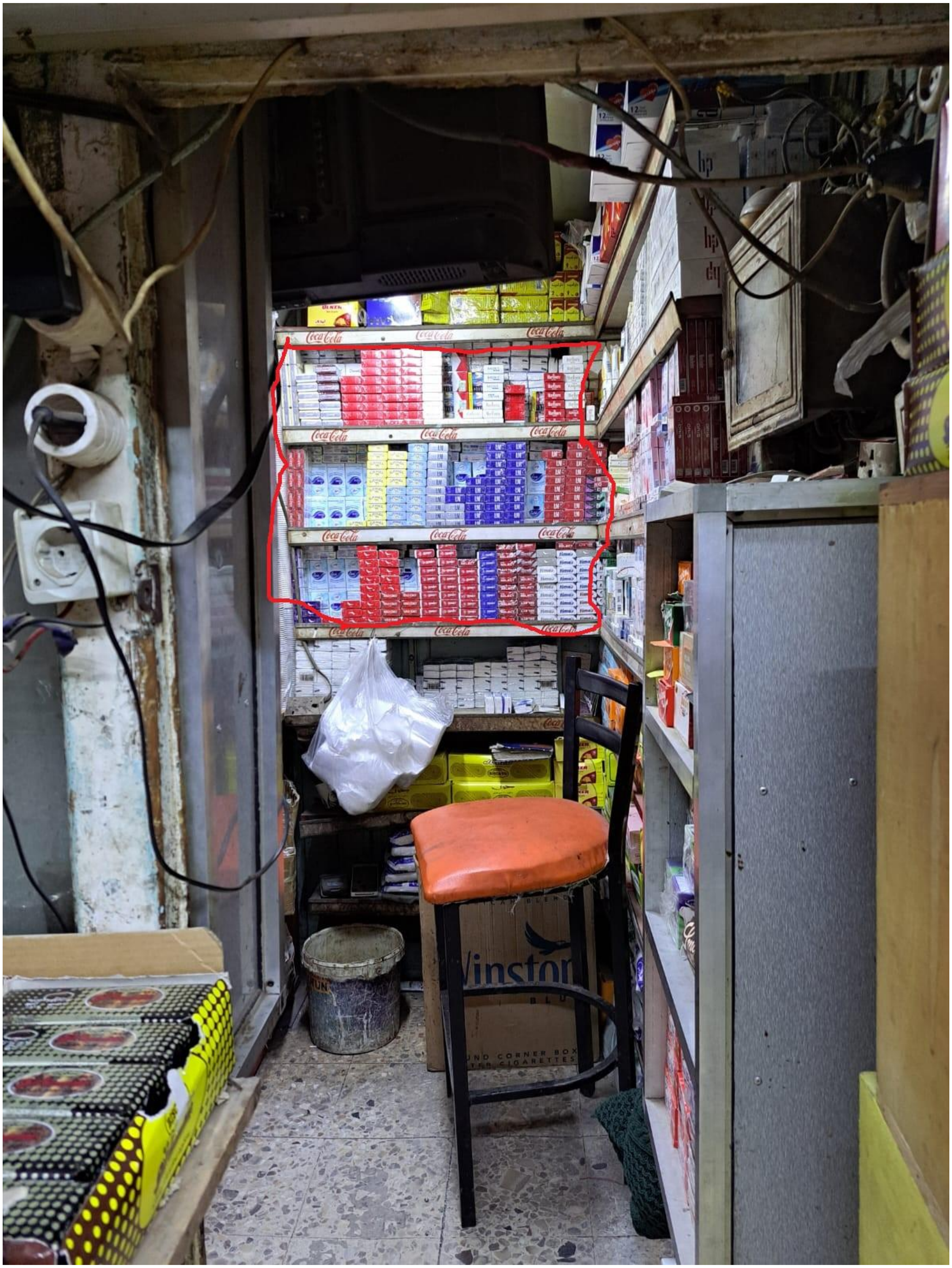

Figure 7 Tobacco products displayed in a street kiosk in Cairo - MARKED

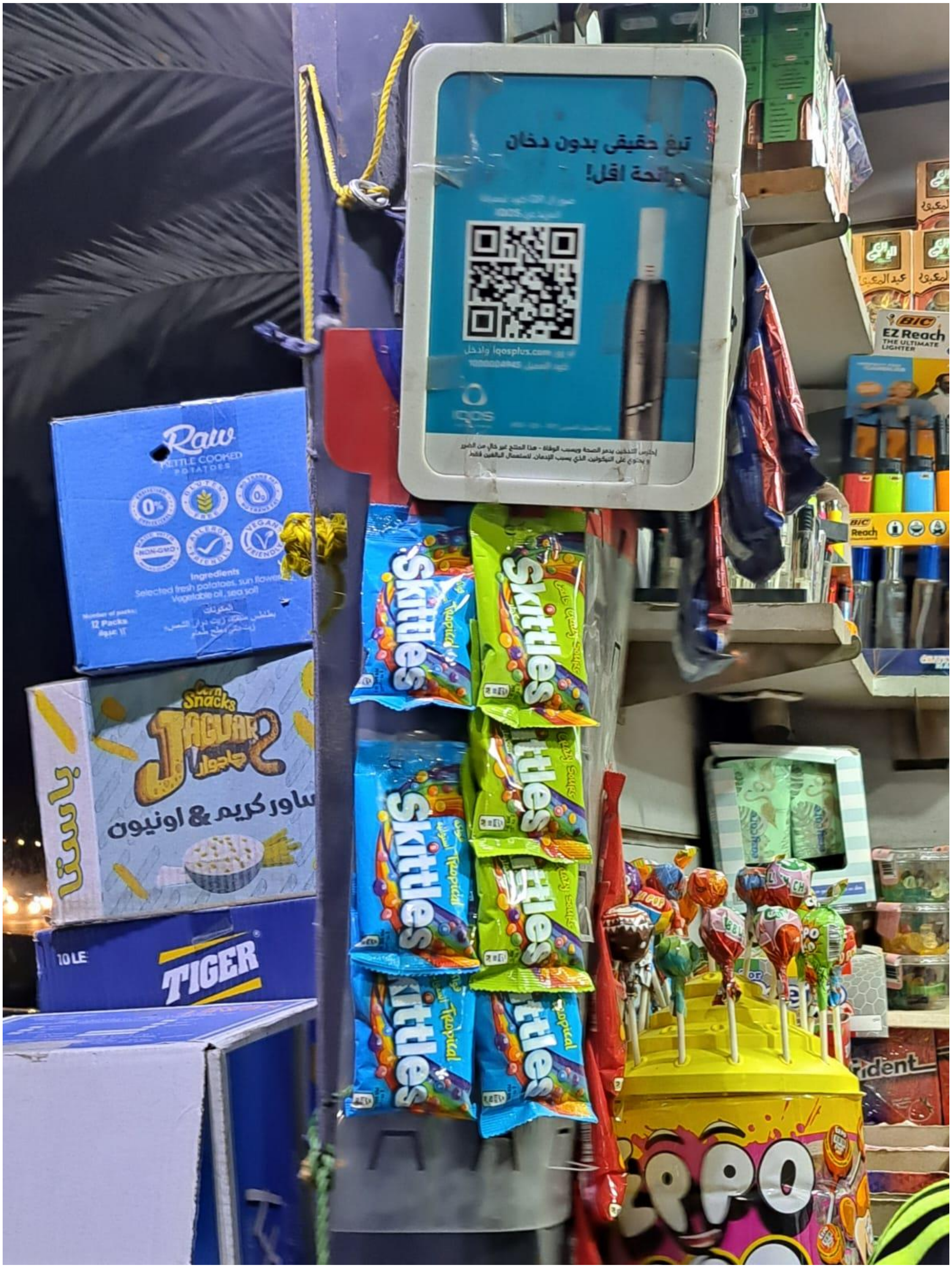

Figure 8 Branded advertisement of IQOS: “True tobacco, smokeless, with less smell!”

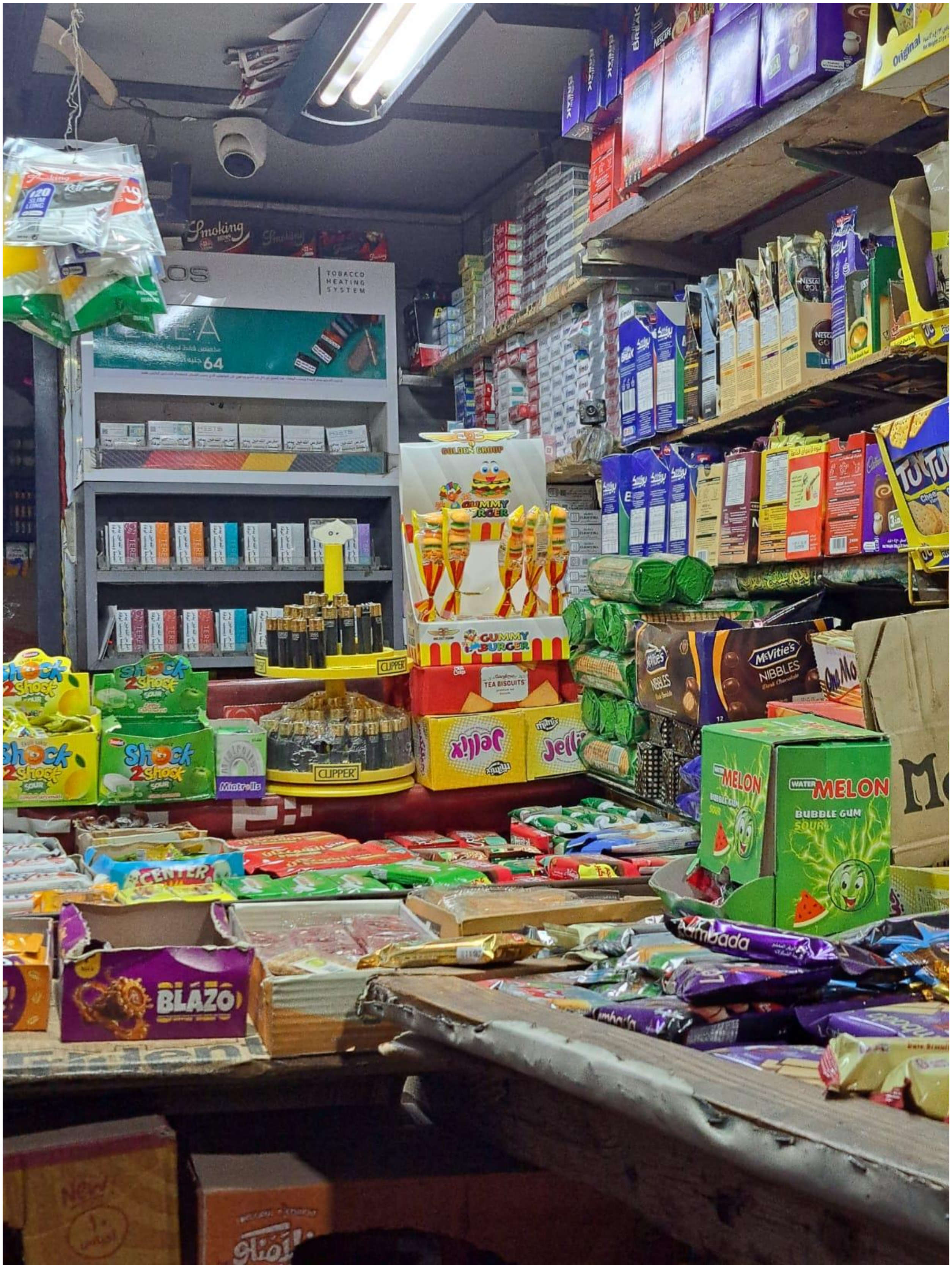

Figure 9 Display of HTP and placement near candy at street kiosk in Giza

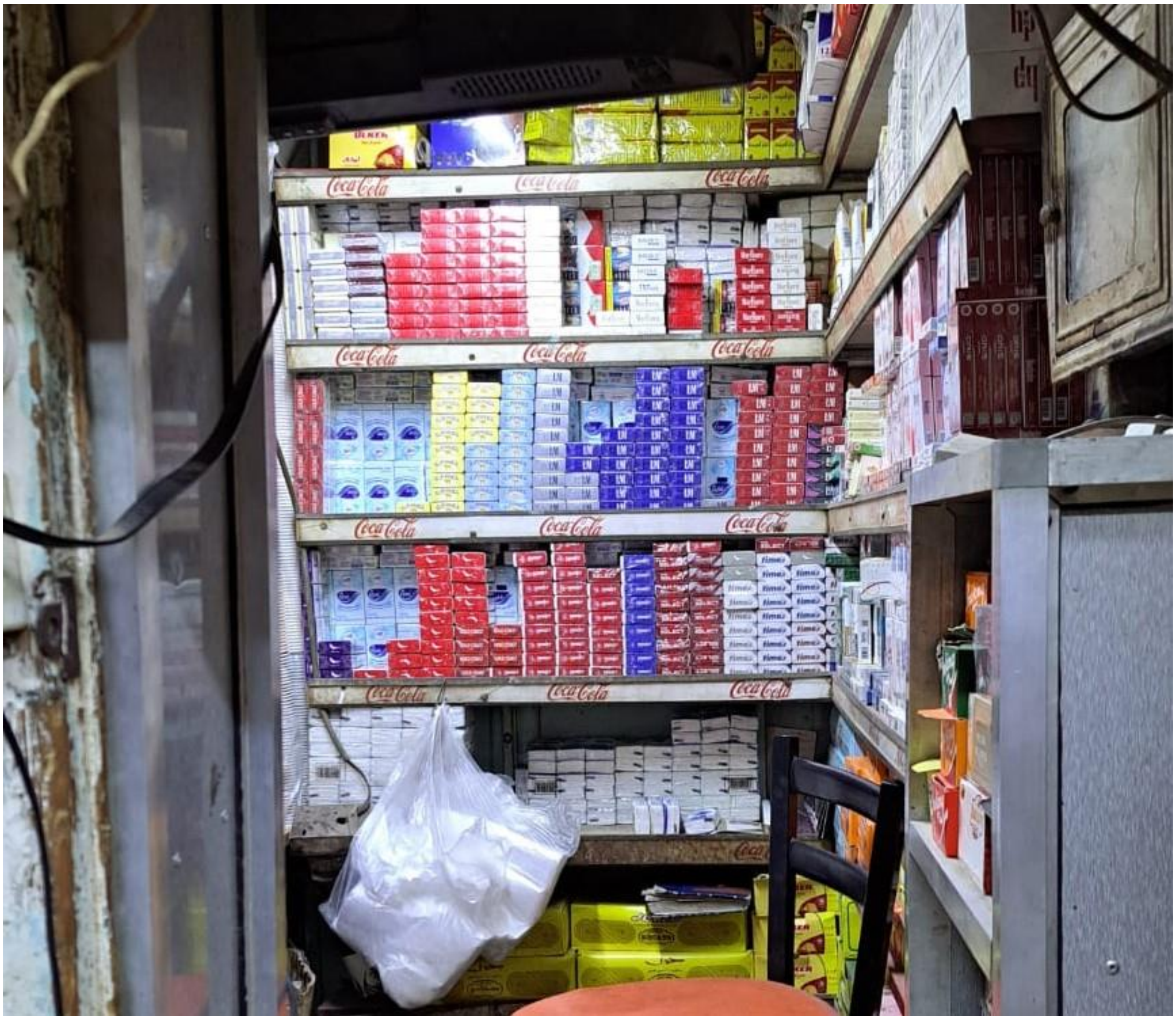

Figure 10 Tobacco products displayed in a street kiosk in Cairo

© 2025 Haridy A. et al.
